# Clinical particularities and response to the anti-inflammatory effect of antiviral treatment in patients with Chronic Hepatitis C and Rheumatoid Syndrome

**Published:** 2012-12-25

**Authors:** GC Lilea, CT Streba, CL Baloseanu, CC Vere, I Rogoveanu

**Affiliations:** * University of Medicine and Pharmacy, Craiova; ** Research Center of Gastroenterology and Hepatology of Craiova, University of Medicine and Pharmacy, Craiova; *** Department of Gastroenterology, University of Medicine and Pharmacy, Craiova

**Keywords:** HCV, CHC, BMI, CRP evolution

## Abstract

Hepatitis C virus (HCV) is an important cause for the development of serious hepatic disease such as chronic hepatitis and liver cirrhosis. Though the pathological mechanisms are poorly understood, it is well established that CHC plays an important role in several immune mediated conditions, including rheumatoid arthritis. We focused on the clinical particularities of patients with CHC and associated RS and we specifically investigated the anti-inflammatory role of IFN-alpha concerning the rheumatic symptoms.

## Introduction

Hepatitis C virus (HCV) is an important cause for the development of serious hepatic disease such as chronic hepatitis and liver cirrhosis [**1**].

 Though the pathological mechanisms are poorly understood, it is well established that CHC plays an important role in several immune mediated conditions, including rheumatoid arthritis.

Clinical particularities of patients with both CHC and RS can help identifying these patients as early as possible. A correct diagnose is needed especially due the complicated management of these patients as many drugs used for the rheumatic manifestations, including corticosteroid and immunosuppressive medication, can be harmful for the liver status [**2**]. 

 It is well known that the interferon-alpha in association with ribavirin has an important impact regarding the treatment of chronic CHC but interferon also has a worth-investigating inflammatory effect that might positively influence the rheumatologic manifestations associated with CHC.

 We focused on the clinical particularities of patients with CHC and associated RS and we specifically investigated the anti-inflammatory role of IFN-alpha concerning the rheumatic symptoms.

## Patients and Methods

In our study, we investigated the response to antiviral therapy for a group of 25 patients with both CHC (genotype 1) and RS. The disease duration was 7-10 years for CHC and 3-5 years for RS since the moment of diagnose. These patients took part in our study during their admission to the Department of Gastroenterology of the Emergency County Hospital of Craiova between 2010 and 2012. 

 Inclusion criteria were: 

 -patient particularities: age < 65, conclusive anamnesis data

 -liver particularities: detectable viral load and minimal levels of liver cytolysis enzymes

 -rheumatologic particularities: negative rheumatoid factor and anti-citrullinated peptide (anti-CCP) antibodies; minimal elevation of serological inflammation markers; absent radiological modifications; limited arthralgia and morning stiffness. 

These 25 patients received 180 micrograms Peg-IFNα2a and 800-1000mg/day of ribavirin according to their body weight, for a period of 12 months. All patients also used intermittent medication for pain relief such as non-steroidal anti-inflammatory drugs for limited periods of time according to the intensity of their symptoms. 

 We statistically evaluated the anamnestic particularities of the patients with CHC and RS and also, we investigated the evolution of the inflammation markers -erythrocyte sedimentation rate (ESR), C-reactive protein (CRP)- at the beginning of treatment, 3 months later and at the end of therapy by reviewing their medical charts.

## Results

**Fig. 1:**
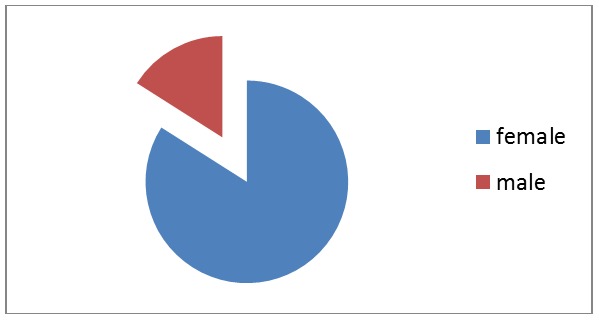
Gender distribution

 The group of patients diagnosed with both CHC and RS consisted of 21 female patients and 4 male patients (**Fig. 1**).

This distribution shows that the association between CHC and RS occurs more frequently in female patients (84%). 

**Fig. 2:**
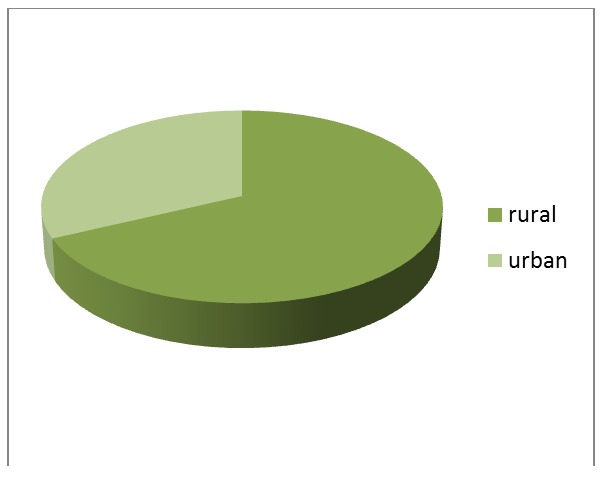
Environment distribution

The medical charts indicated the fact that 68% of the selected patients come from the rural environment (**Fig. 2**). 

**Fig. 3:**
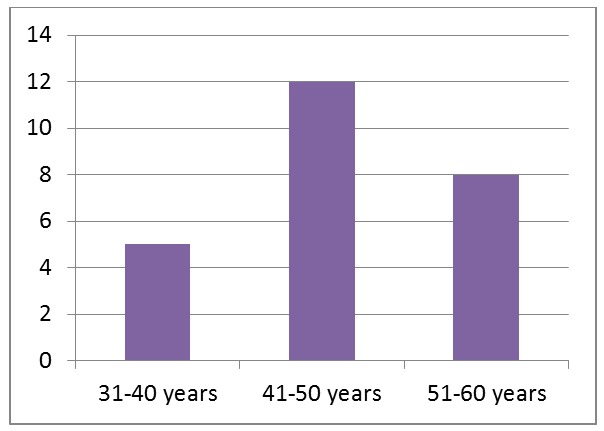
Age distribution

 Regarding the age range, all patients were aged 30 to 50. Five patients (20%) belong to the 4th decade, 12 patients (48%) belong to the 5th decade and finally, the 6th decade includes 8 patients (32%) (**Fig. 3**). The age related distribution shows the predominance of the patients belonging to the 5th decade of life.

**Fig. 4:**
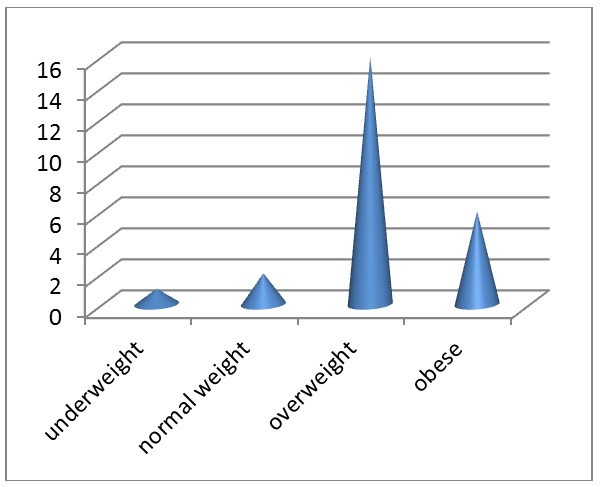
BMI distribution

The results of the analysis of body mass index concerning the patients in our study are shown in **Fig. 4**. Thus, 66% of the patients had a normal weight, 24% were overweight, 6% were obese and 4% were underweight. 

The body mass index distribution indicates the predominance of normal weight among selected patients (66%).

Regarding the rheumatic manifestations, all patients presented:

- symmetrical arthralgia concerning maximum 3 joints, of various intensity (minimal, medium, high); 

 - morning stiffness for less than 30 minutes;

 - functional disability regarding each affected joint (example: plantar flexion, shoulder rotation) 

**Fig. 5:**
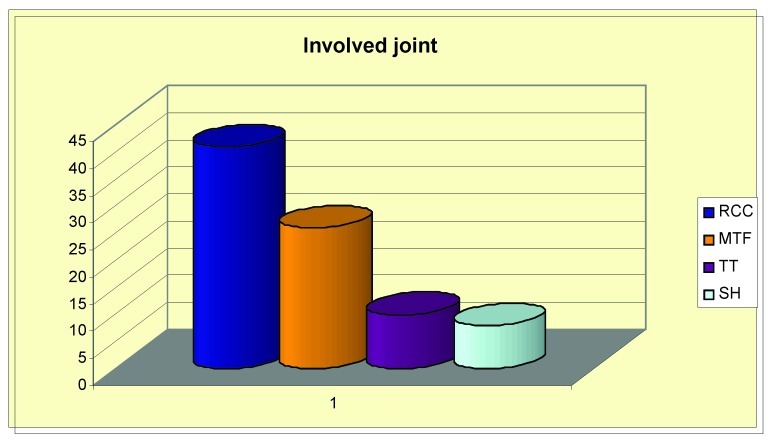
Distribution of the involved joint

 The analysis of the type of affected joint is presented in **Fig. 5**.

 Thus, 22 patients (88%) patients had arthralgia concerning the radiocubitocarpien joint, the metatarsophalangien joint was interested in 14 patients (52% ), the tibio-tarsul joint was interested in 6 patients 24% of patients and 8 patients (8%) had artralgia concerning the scapulohumeral joint. 

The analysis of the inflammation syndrome led to clinical and serological results related to the use of Peg-IFNα2a –ribavirine therapy. **Tables 1** and **2** show the evolution of ESR and CRP during antiviral therapy.

**Table 1:** ESR evolution. ANOVA p-value < 0.0001 Highly signifiant difference

ESR	Start	3 months	12 months
Mean	52,680	34,200	19,320
Standard deviation	6,216	7,583	2,512
C.V.(%)	11,80%	22,17%	13,00%

In all 25 patients treated with Peg-IFNα2a –ribavirine, we observed that ESR and CRP registered a decrease along the 12 months of therapy. Moreover, anamnestic data at the end of the follow-up period indicated absent arthralgia, morning stiffness and functional disability.

**Table 2:** CRP evolution. ANOVA p-value < 0.0001 Highly signifiant difference

ESR	Start	3 months	12 months
Mean	14,560	11,800	5,141
Standad deviation	3,176	2,466	0,635
C.V.(%)	21,82%	20,90%	12,35%

## Discussion

Interferon-alpha is famous for its impact upon the treatment of CHC but the role of this therapy in regard to the rheumatologic manifestations associated with CHC has only recently started to be investigated. 

 In this article, we investigated 25 patients with documented RS and CHC who were submitted to a 12-month therapy of Peg-IFNα2a/ribavirin and the results showed that articular symptoms and serological inflammation markers decreased. 

 Moreover, the common clinical particularities of the selected patients were studied in order to better understand the clinical profile of patients with CHC and RS (**Table 3**).

**Table 3:** Clinical particularities

Gender distribution	Male 16%	Female 84%		
Life decade distribution	31-41 years old 20%	41-51 years old 48%	51-61 years old 32%	
Enviorment distribution	Rural area 68%	Urban area 32%		
BMI	Number pacients			
Normal weight	16 (64%)			
Overweight	16 (24%)			
Obese	2 (6%)			
Underweight	1 (4%)			
Affected joint	RCC joint 88%	MTP joint 52%	MTP joint 52%	SH joint 8%

This distribution regarding the female/male gender shows that the association between CHC and RS occurs more frequently in female patients (84%).

The results match the medical literature data as rheumatic manifestations are acknowledged to be more frequent in women [**1**].

The age related distribution shows the predominance of the patients with CHC and RS belonging to the 5th decade of life.

On the other hand, studies indicate a higher incidence of rheumatoid arhtritis in patients belonging to the 4-5th decade of life but they also show that CHC is more frequent in the 6th decade of life [**1**]. 

The environment distribution indicated the fact that 68% of the selected patients come from the rural environment as opposed to the patients from urban area (32%). One possible explination for this higher rural frequency of patients with CHC and RS could be the difficult access of persons to medical services and also, the greater exposure to the risk factors of both related diseases. 

The body mass index distribution indicates the predominance of normal weight among selected patients.

Body mass index was taken into consideration due to the fact that negative factors for the prediction of sustained virusological response include a high body mass index for various reasons [**2**]:

- decreased immunity 

- occurance of steatosis that reduces the response to interferon 

- negative impact on the pharmacokinetcs of interferon 

The clinical features of the CHC associated rheumatoid syndrome often resamble reumatoid arthritis [**3**]: 

- symmetrical arthralgia in small joints

- morning stiffness

- functional disability

In our study, clinical rheumatic features were observed in all patients during each medical check-up in order to evaluate the rheumatoid syndrome and to compare its evolution during antiviral therapy. All patients experienced typical rheumatic inflammatory manifestations at the initial moment of our study including: symmetrical arthralgia concerning maximum 3 joints, of various intensity (minimal, medium, high); morning stiffness for less than 30 minutes; functional disability regarding each affected joint (example: plantar flexion, shoulder rotation). 

The anlysis of the type of affected joint showed a greater impact of the RS on the radiocubitocarpien joint (88%), followed by the metatarsophalangian joint. Researchers affirm that there are two distinct forms of rheumatic syndrom which is associated to CHC: 

- the first form respects the symmetry of the joint involvement and resambles the clinical profile of rheumatoid arthitis. Rheumatoid factor can be positive but the anti-CCP atibodies remain negative so they can separate the two diagnoses. 

- the second form involves one or very few joints, especially large and medium ones [**4,5**].

In our study, the rheumatoid syndrome combines the two forms above, mentaining the joint symmetry, a small number of affected joints and involving especially medium joints. 

In all 25 patients treated with Peg-IFNα2a –ribavirine, we observed that ESR and CRP registered a decrease along the 12 months of therapy. Moreover, absent arthralgia, morning stiffness and functional disability were noticed at the end of the follow-up period and we suspect that the anti-inflammatory effect of the interferon is responsible for the improvement of these rheumatic features.

Certain studies focused on the immunoregulatory and anti-inflammatory properties of IFN-alpha suggesting a variety of mechanisms through which this drug might reduce the inflammatory process [**6-9**]:

- the ability of the IFN-alpha to reduce IL-1 

- down-regulation of pro-inflammatory gene products

- the induction of IL-10 

- elevated response from the hypothalamic-pituitary-adrenal axis

In our study, the anti-inflammatory and immunoregulatory activity of the IFN-alpha is proven by the down-regulation of the inflammation marker levels and the benefit to the rheumatic symptoms for all patients that underwent this type of therapy. 

## Conclusions

We statistically evaluated the common anamnestic particularities of 25 patients with CHC and RS who initiated treatment with Peg-IFNα2a–ribavirine, which are important in order to better understand the clinical profile of patients with CHC and RS for an early diagnose and treatment onset.

Also, we investigated the evolution of the rheumatic symptoms and of the inflammation markers -erythrocyte sedimentation rate (ESR), C-reactive protein (CRP)- at the beginning of treatment, 3 months later and at the end of therapy by reviewing their medical charts. 

The majority of patients in our study were women, mainly from the rural area and almost half of them were in their fifth decade of life. Body mass index varied but we mainly encountered normal weight patients.

All patients experienced typical rheumatic inflammatory manifestations at the initial moment of our study including: symmetrical arthralgia concerning maximum 3 joints, of various intensity (minimal, medium, high); morning stiffness for less than 30 minutes; functional disability regarding each affected joint (example: plantar flexion, shoulder rotation). The pattern of the RS for our selected patients mentained the joint symmetry, affected a small number of joints and involved especially medium joints. 

We focused on the inflammation process included in the pathology of CHC-related rheumatoid syndrome and also on the anti-inflammatory function of IFN-alpha that was the basic treatment for our patients. 

The results of our study show that, when used in the treatment of patients with CHC and associated rheumatoid syndrome, Peg-IFNα2a anti-inflammatory activity can improve the rheumatic manifestations by down-regulating the inflammation markers and reducing the rheumatic inflammatory symptoms.
